# Several routes of cell death to secondary necrosis in the elasmobranch testis

**DOI:** 10.1007/s10495-022-01733-0

**Published:** 2022-06-07

**Authors:** Leon Mendel McClusky

**Affiliations:** Anatomy Section, Department of Health & Care, Faculty of Health Sciences, UiT The Arctic University of Norway, Campus Narvik, Narvik, Norway

**Keywords:** Spermatogonial apoptosis, Secondary necrosis, PCNA, Phagocytosis, Sertoli cells

## Abstract

The process of spermatogenesis features significant germ cell loss through apoptosis. Routine histology of the testes of well-studied animal models hardly discloses any trace of their phagocytic clearance by the supporting Sertoli cells. This review highlights lessons learnt from the cystic, diametric testes of some seasonally migrating elasmobranchs (e.g., spiny dogfish and blue sharks) that offer unconventional investigative paradigms to study these phenomena as these organs readily disclose a pronounced apoptosis gradient affecting exclusively spermatogonial clones that each are enclosed with their own Sertoli cells in spherical structures called spermatocysts. This gradient is visible at a certain time of year in the spermatogenically active shark, and peaks in mature spermatogonial cysts as clustered deaths with sporadic, and not massive secondary necrosis. Conversely, immature spermatogonial cysts in blue sharks reveal a characteristic periluminal display of single apoptotic deaths. Tracing aberrations in the immunostaining patterns of the conserved cell cycle marker, proliferating cell nuclear antigen, the gradual progression of the death process in individual or coalesced spermatogonia in contiguous cysts becomes clear. The multiple apoptotic nuclear fragmentation morphologies inform also of a protracted death process involving three different morphological routes of nuclear fragmentation (of which some are TUNEL-positive and other TUNEL-negative) and concomitant chromatin compaction that culminate in freed apoptotic bodies (i.e., secondary necrosis). It is discussed that the staggered spermatogonial deaths and accompanying intermittent secondary necrosis in mature blue shark spermatogonial cysts may well relate to the low phagocytosis capacity of cyst’s Sertoli cells that are still functionally naïve.

## Introduction

In the testis, immature spermatogonia undergo a species-specific number of mitotic divisions before they proceed through meiosis and give rise to round spermatids that ultimately transform into spermatozoa. These maturational developments, known as the process of spermatogenesis, is self-sustaining and self-adjusting. Studies of the tubular testicular parenchyma of the common laboratory rodent reveal that the sperm output resulting from the base-to-lumen development of the germ cells in the epithelial wall occur with a lower-than-expected efficiency [1, 2], with sporadically degenerating germ cells mainly implicated [3–5].

The incidence of germ cell degeneration in the mammalian seminiferous tubule is variable, with some stretches of the tubule (i.e., defined groupings of germ cells called stages) revealing only one pyknotic cell per 200 tubules examined [5]. However, not all germ cell deletions may present as frank deaths since the deletion of immature spermatogonia in the rat tubules is even described as morphologically inconspicuous [6]. It is also thought that dead germ cells are rapidly and efficiently eliminated via phagocytosis since routine histology of the tubules reveals no trace of their phagocytosis [7–9]. The phagocytes within the tubules are supporting somatic cells, called Sertoli cells, that each cradle 30–40 germ cells in various stages of differentiation in its tree-like cell body [10]. The latter report estimates that Sertoli cells clear millions of apoptotic germ cells during spermatogenesis. It is clear, however, that maintenance of an orderly tissue architecture that is conducive for cellular production and germ cell differentiation is an abiding characteristic of the normal testis.

**Fig. 1:**
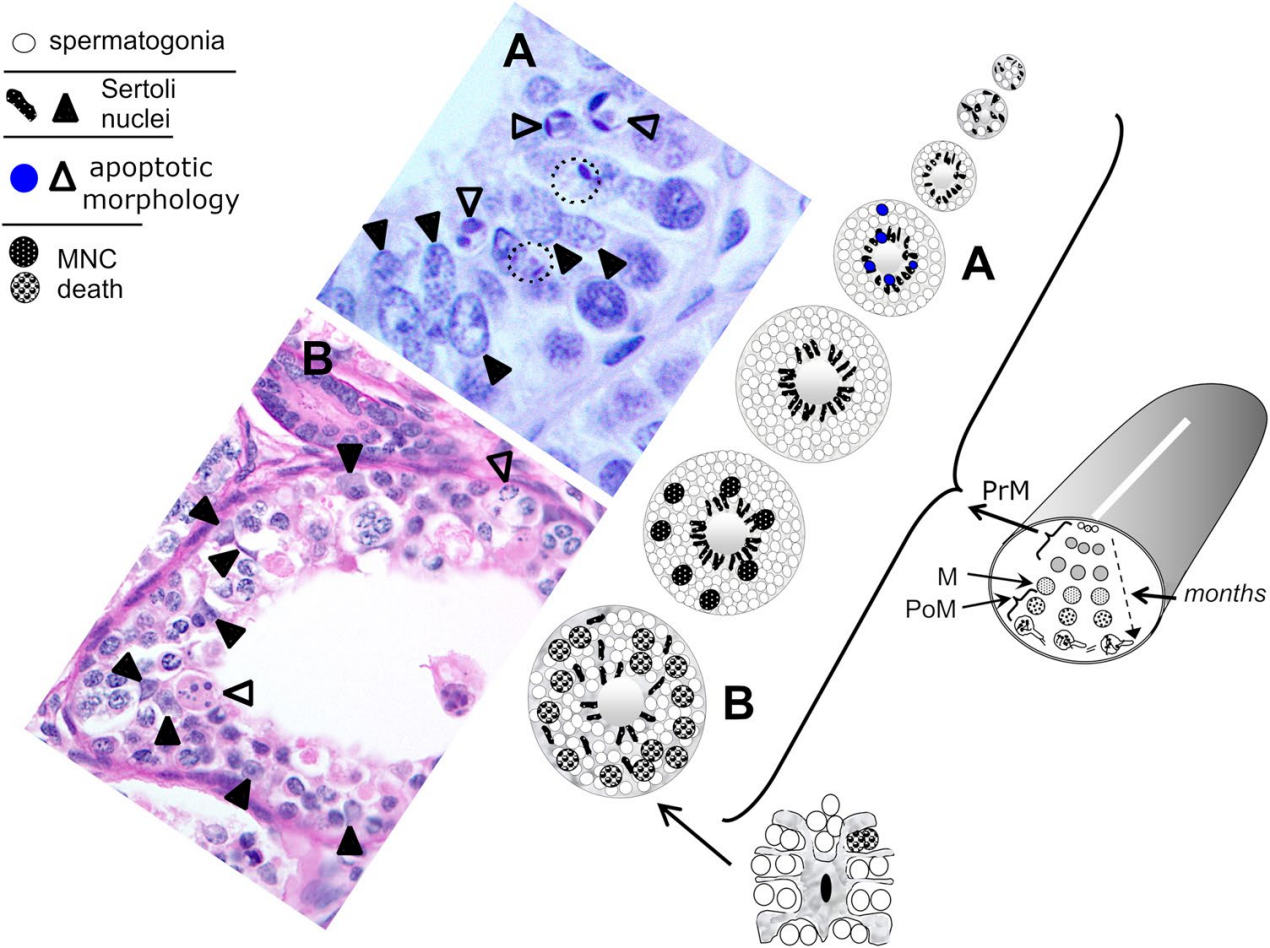
Schematic illustration of the topographical arrangement of successive spermatocyst stages in a cross-sectional view of the blue shark testis, showing the apoptosis gradient that characterizes the PrM region in spermatogenically active blue sharks**.** Histologically this gradient presents as extensive MNC that peaks in mature PrM cysts (**B**). In contrast apoptotic phenomena in smaller PrM cysts (**A**) have a decidedly periluminal character showing crescents of compacted chromatin and pyknotic spots (open arrowheads) amongst the Sertoli cell nuclei (filled arrowheads). Adjacent round bodies (circles) only just reveal the last remains of these crescents, indicating the transient nature of these morphologies. *M* meiotic cysts (i.e., primary, and secondary spermatocytes), *PoM* postmeiotic cysts (i.e., round, maturing and fully differentiated spermatids) (Colour figure online)

How such diametrically opposite cellular processes can proceed seamlessly during normal spermatogenesis in vertebrates has been the subject much enquiry, including in the study of cystic spermatogenesis in unconventional animal models, such as elasmobranchs (e.g., sharks). The testes of the latter and other anamniotes consist of anatomically discrete, follicle-like spermatocysts, each containing a specific intercellular bridge-connected germ cell stage (i.e., isogeneic clone) and its own complement of Sertoli cells. The testes of some elasmobranch species (e.g., spiny dogfish and blue shark) offer another advantage in that maturing cysts occur in succession across the testicular diameter (Fig. 1). It should also be kept in mind that because spermatogenic development proceeds in a wave-like, linear fashion in contiguous cysts, cysts immediately upstream may hold the clues to earlier stages of any given developmental process that have reached an advanced stage in cysts downstream. The life–death balance in these cytoplasmically linked germinal clones in the elongated diametric testis of seasonal breeding elasmobranchs (e.g., spiny dogfish and blue shark) has attracted much interest due mainly to the ready discrimination of the apoptosis-susceptible germ cell stages, owing to a premeiotic (PrM) stage-related apoptosis gradient that is visible in a single testicular cross-section at a certain time of year in the spermatogenically active individual (Fig. 1). The gradient peaks in mature spermatogonial cysts, manifesting as extensive multinucleate cell death (MNC). In contrast, a subset of immature spermatogonial cyst stages in the blue shark display a periluminal pattern of individual classic apoptotic morphologies, including crescents of compacted chromatin and pyknotic bodies. The aim of this review is to summarize particularly the novel observations of testicular apoptosis in the blue shark, which are evidence of different morphological routes of nuclear fragmentation and concomitant chromatin compaction that culminate in the phenomenon of scattered, instead of massive secondary necrosis, even in the fully condemned PrM cyst. The characteristic periluminal display in the blue shark testis of multiple isolated deaths in immature PrM cysts are routine observations in the same cysts in free-ranging blue shark sampled from both the Northwest Atlantic [11, 12] and Mediterranean populations sampled at Bari, Italy (unpublished observations), suggesting that it is likely a species-specific phenomenon.

The slowly progressing MNC death and subsequent secondary necrosis in condemned spermatogonial clones during normal spermatogenesis in elasmobranchs are curious exceptions to accepted dogma about the maintenance of tissue integrity and homeostasis. It is generally thought that the apoptotic cell is, as a general rule, cleared swiftly by phagocytes, a process commencing already in the earliest stages of apoptotic death while the plasma membrane is still intact such as to prevent the release of noxious cellular contents that may occur at the secondary necrosis phase [10, 13]. It has, however, also been countered that secondary necrosis during late apoptosis is the natural outcome of fully developed apoptosis in animals [14]. This argument is, in turn, rooted in observations that in vivo secondary necrosis occurs, among others, typically in tissues with ducts and lumina during which the apoptotic bodies from the dying epithelial cells are shed into the ducts and lumina, developments presumably due to delayed, non-availability or lower efficiency of phagocytosis by non-professional (specialized) phagocytes [14]. The question is therefore why apoptotic death during elasmobranch spermatogenesis is not swift, but rather protracted and associated with some incidences of secondary necrosis.

## Multiple modalities of death inform about systematic nuclear fragmentation

Spermatogonia are patently the cell type undergoing cell death in the wholly condemned mature PrM cyst of the blue shark (Fig. 1) and in dogfishes [15, 16]. A similar frank inference regarding the observed periluminal apoptosis in the blue shark’s small PrM cysts seems much less straightforward in routine histology given their proximity to the Sertoli nuclei. Moreover, such a pattern of death is never seen in the spiny dogfish [17]. The simpler organization of the elasmobranch testis renders it suitable for more sensitive methods to explore the underlying molecular aspects of this cell death and simultaneously verify the cell type involved in the periluminal display of apoptosis. The fragmentation of DNA was the first to be characterized as a biochemical hallmark of apoptotic death [18] and subsequently linked to chromatin condensation morphologies [19]. One conventional assay to assess DNA fragmentation in situ is the terminal dUTP nick end-labeling (TUNEL) method that preferentially detects low molecular weight DNA fragmentation that is the predominant type of fragmentation in apoptotic deaths [20, 21].

**Fig. 2:**
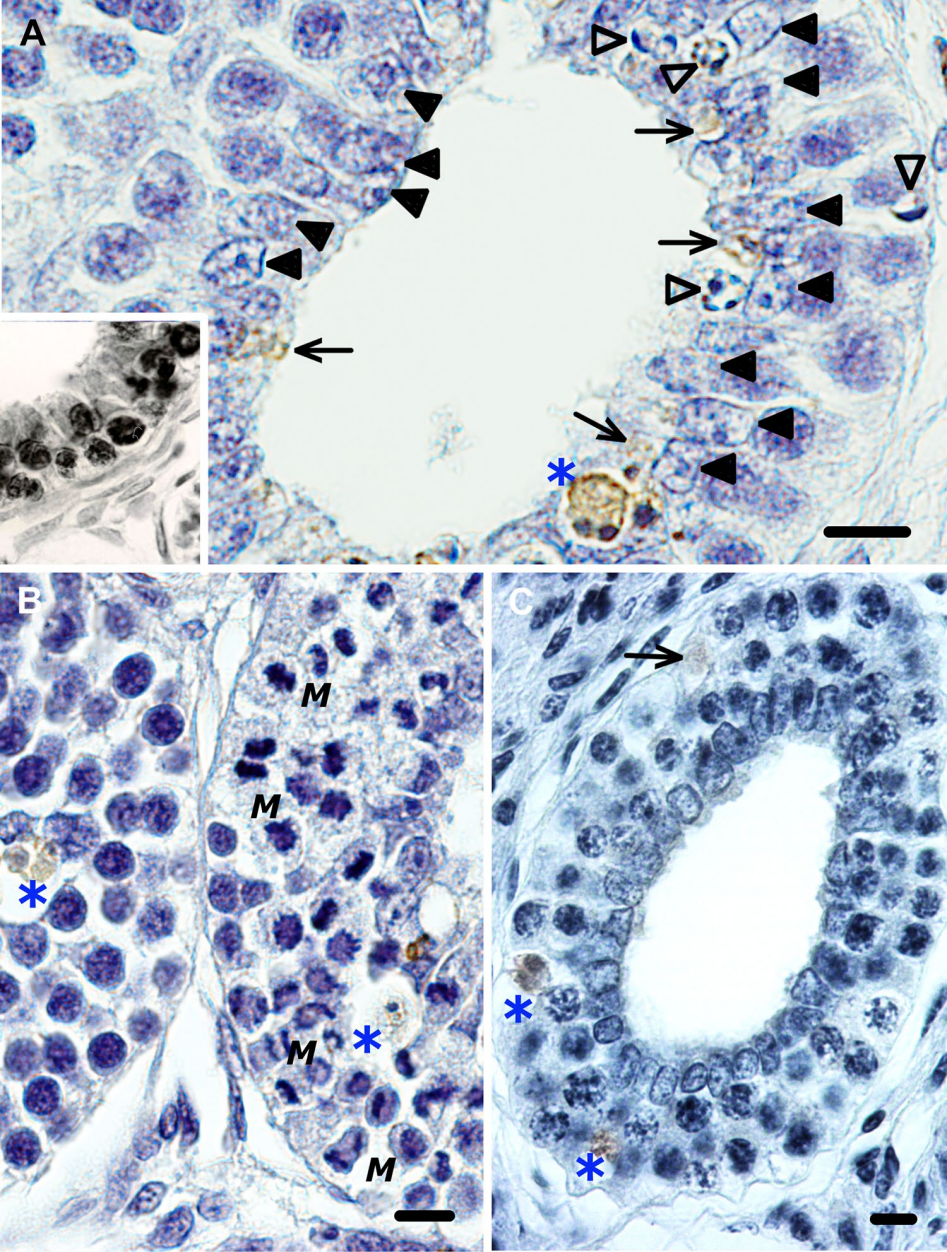
Comparison of TUNEL-staining patterns of developing PrM cysts in some elasmobranch species. **A** TUNEL staining of small blue shark PrM cysts also reveal a periluminal character, except for some classic apoptotic morphologies (open arrowheads) that are not TUNEL-labeled. A scattering of subcellular (small arrows) and cell-sized (asterisk) bodies, with or without pyknotic spots, are crisply TUNEL-stained. Inset: On the rare occasion that it is detected with the technique, the entire spermatogonial clone in the corresponding cyst stage in the spiny dogfish is TUNEL-positive, a sight never encountered in the blue shark testis. TUNEL-staining may label a scattering of spermatogonia in mature PrM cysts of the **B** blue shark and **C** thresher shark, but notably spermatogonia that display one or two pyknotic spots (asterisks). Bar = 10 µm (Colour figure online)

As shown in Fig. 2, the pattern of TUNEL staining in the smaller PrM cysts of the blue shark largely mirrors the periluminal pattern seen with routine histology. However, close inspection of the periluminal TUNEL staining reveals that the ring-like perinuclear compacted chromatin and crescents of chromatin are distinctly TUNEL-negative. Instead, periluminal cell remnants of various sizes, with or without pyknotic spots, are TUNEL-labeled. The odd occurrence of a TUNEL-negative nucleus on the cyst periphery, with crescents of condensed chromatin clearly confirms these phenomena as spermatogonial deaths. Total TUNEL-labeling of the entire spermatogonial clone, as seen very sporadically in the spiny dogfish, is never encountered in the blue shark. TUNEL-positive spermatogonial corpses showing one or two pyknotic masses are also consistently observed in larger PrM cysts of the blue shark (Fig. 2B) and thresher shark (Fig. 2C).

The differential staining with the TUNEL method is meaningful when these observations are considered in the light of the two-step process of DNA fragmentation during apoptosis. The first phase is high molecular weight (HMW) that is TUNEL-negative and that is usually, but not always, followed by internucleosomal cleavage of DNA that generates the shorter typically TUNEL-detectable double-stranded breaks of these nucleosomal fragments [20, 22]. Systematic analyses of the course of experimentally induced apoptotic death in vitro have determined that HMW fragmentation, is coincident with the early stages of chromatin condensation (termed stage I), namely the collapse of chromatin outward against the nuclear periphery, also termed perinuclear chromatin compaction [22]. Conversely, nucleosomal fragmentation is associated with the subsequent advanced (stage II) chromatin condensation, namely the formation of pyknotic or apoptotic bodies [23, 24]. Thus, assuming that these molecular mechanisms are conserved, then the differential TUNEL labeling observed here during blue shark spermatogenesis has additionally highlighted what appears to be a protracted process of apoptotic death during spermatogenesis in these primitive vertebrates.

The periluminal apoptotic morphologies in the developing PrM cyst in the blue shark clearly indicate that it is single spermatogonia that exit the cell cycle into apoptosis. However, TUNEL immunohistochemistry is seemingly not sensitive enough to detect earlier signs of imminent entry into death in the peripheral layers of cycling spermatogonia. A previous quantitative analysis of cell death and proliferation in the blue shark testis [12] surprisingly reported an overlap between immunolabelling of the periluminal apoptotic morphologies with TUNEL and that of the conserved cell cycle marker, proliferating cell nuclear antigen (PCNA). However, PCNA immunohistochemistry also anomalously labeled the odd clone member in otherwise appropriately immunostained spermatogonial clones. With formalin-fixed samples typically the primary material for these analyses in wild-caught species, the properly analyzed elasmobranch testis nevertheless offers interesting investigative paradigms for the study of the waxing and waning of the immunoexpression of a conventional cell cycle marker such as PCNA in a spherical clone, and whose expression is absent during M-phase. PCNA is a conserved auxiliary protein to DNA polymerases δ and ε in the replication fork [25, 26]. It is generally agreed that its immunohistochemical staining intensity in situ reaches peak levels in late G1 and for the duration of S-phase after which its steadily diminishes through G2-phase to non-detectable levels during mitotic division [27–29].

These landmarks have been mapped in cycling immature spermatogonial clones in the blue shark [30]. Briefly, by using the intense dark brown staining pattern (indicating late G1‐S-phase) and the PCNA-negative mitotic figures as definitive “signposts” in a spherical cycling clone (i.e., the cyst), it was determined that the protracted passage through the four phases of the cell cycle manifested as variations in the immunoexpression of PCNA in the different clone members. Accordingly, clones or clone members in G2-phase were uneven light brown stained/globular-like). The fortuitous sight of occasional clones engaged in the S-phase → G2 transition confirmed the uneven light brown/ globular appearance as depicting G2. The latter immunostaining pattern was the dominant pattern seen in multiple layers of cysts in the MNC testis condition. Thus, cell-cycle active blue shark spermatogonial clones may either promptly proceed from S-phase into G2 and M-phase, or they may become synchronized in only G2-phase (see Fig. 9 in McClusky [30]).

## More than one route of apoptotic nuclear fragmentation

**Fig. 3:**
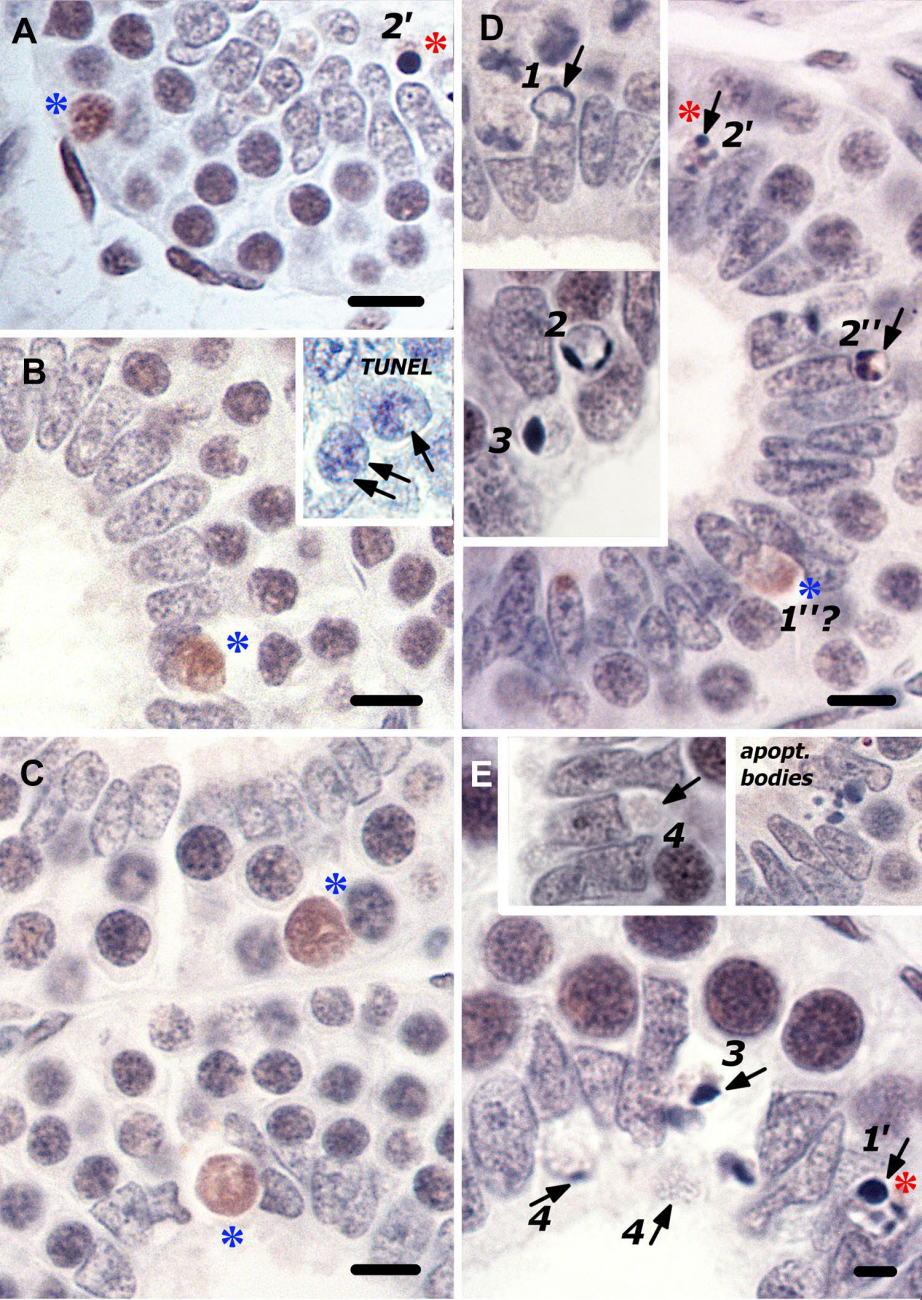
A photomontage of the isolated anomalously PCNA-labeled dying spermatogonia and the various periluminal apoptotic morphologies in otherwise normal two- to three-layered PrM cysts. **A** Although normal looking, this eccentric spermatogonium with a striking globular appearance (blue asterisk), is probably the earliest sign of entry into death while the rest of the clone is about to exit S-phase. **B** A shrunken, strongly orange PCNA-labeled spermatogonium (blue asterisk) displays noticeably fewer but larger “globular areas”. (Inset) Spermatogonia with large clear areas (arrows) are unlabeled in TUNEL-stained sections. **C–E** The anomalously PCNA-labeled spermatogonia (blue asterisk) that are often ensconced amongst the Sertoli cell nuclei, increasingly display a PCNA-deficient region that may or may not reveal bluish cytological detail. Some of these oddly PCNA-labeled deaths are coincident with the periluminal apoptotic deaths whose various stages of progression seem to indicate three different morphological routes of nuclear fragmentation and concomitant chromatin compaction that culminate in freed apoptotic bodies (secondary necrosis): route 1 → 4, PCNA-negative progressive perinuclear compaction of chromatin into crescents in an otherwise definitive, opaque nucleus. The crescents of chromatin gradually diminish, leaving only the opaque nuclear remnant at the luminal border (4*,* see also Fig. 1): route 1′ → 2′, weakly PCNA-labeled wholesale fragmentation of the nucleus into pyknotic masses of various sizes (red asterisk); route 1″→2″*,* formation of multiple eccentrically placed pyknotic masses next to a large vacuole-like nuclear fragment against a PCNA-labeled background. This apoptotic morphology could possibly represent a further stage of development of this anomalously PCNA-labeled dying spermatogonium (?). Bar = 10 µm (Colour figure online)

Following PCNA immunohistochemistry of formalin-fixed cross-sections, normal looking PrM cysts with two to three layers of spermatogonia reveal the anomalous PCNA-labeling of a scattering of spermatogonia in addition to the periluminal immunostaining pattern, and also in cysts with no periluminal death phenomena (Fig. 3). Given that a few of the oddly PCNA-labeled spermatogonia are also ensconced amongst the periluminal Sertoli cell nuclei, this suggests that the anomalous PCNA-labeling detects earlier stages of spermatogonial death prior to the manifestation of apoptotic chromatin condensation. It should be kept in mind that cysts immediately upstream may hold clues to earlier stages of the apoptotic death process that have reached an advanced stage in cysts downstream. Accordingly, these earlier stages of the death process entail the gradual redistribution of PCNA in a normal looking strongly immunolabeled clone member that has a distinct exaggerated globular appearance (Fig. 3A), followed by an immunomorphology showing a few larger cleared/“globular” areas (Fig. 3B) and eventually the emergence of one large increasingly PCNA-deficient area (Fig. 3C) that may reveal blue cytological detail, most likely chromatin threads (Fig. 3D). Given the strong immunoexpression of PCNA in these isolated deaths, it is argued that this reflects initially the entry into death at the time of exit from S-phase, and PCNA immunoreactivity is retained because of the slow progression of death (see below).

The scattered anomalously PCNA-labeled dying spermatogonia and classic apoptotic morphologies that border the cyst lumen reflect the deaths of single spermatogonia, which is perhaps the closest link between them in this in situ study of highly dynamic processes. Cognizant of the slow progression of apoptotic death in a primitively organized germinal compartment, it may be apparent then that what initially seems chaotic is indeed structured and biologically significant. Closer inspection reveals what seem to indicate three different morphological routes of nuclear fragmentation and concomitant chromatin compaction that culminate in freed apoptotic bodies (Fig. 3D, E). The clearest indication of the existence of more than one route to dismantle the lonesome dying spermatogonium’s nucleus is that which on the surface appears as the PCNA-labeled compaction of the entire nucleus into a single pyknotic mass, and which is not the case, but rather internal nuclear fragmentation (see Fig. 3A, E). This is contra the unlabeled progressive perinuclear compaction of chromatin ultimately as crescents in an otherwise definitive, opaque nucleus (Fig. 3D, E). The latter simultaneously reveals the transient nature of the morphology showing crescents of condensed chromatin, because these gradually diminish, leaving only the opaque nuclear remnant at the luminal border (Fig. 3E). It is the latter large remnant of the nucleus and the weakly PCNA-labeled nuclear fragment with one or two pyknotic spots, that are commonly TUNEL-labeled.

These light microscopic descriptions of nuclear fragmentation during the apoptotic death process in single blue shark spermatogonia strongly resembles the description of multiple pathways of apoptotic nuclear fragmentation and ensuing apoptotic bodies observed in experimentally induced apoptosis in myelomonocytic tumor cells [31]. Moreover, the morphology showing the large vacuole-like area and eccentrically placed small pyknotic spots (see Fig. 3D) strongly resembles that described as nuclear fragmentation by budding in apoptosing thymocytes [32].

It is reasoned that the anomalous PCNA-labeling of normal-looking spermatogonia reflects the genuine detection of this nuclear factor and its putative involvement in altered nuclear architecture very early in the commencement of the death process and this would agree with the protracted or intermittent rates of cellular processes at this phyletic level. The disassembly of the lamins, one of a number of structural components of the nuclear scaffold or matrix, is thought to be essential for the successful completion of nuclear apoptosis [33]. Their degradation in apoptosis in several cell types is concomitant with the significant accumulation of a number of proteins in the nuclear matrix fraction, including PCNA [34]. The latter authors have argued that the retention of the latter proteins probably alludes to the requirement of an elementary protein scaffold for the process of chromatin compaction. Interestingly, another school of thought asserts that the nuclear matrix is markedly affected during apoptosis, based on the development of a large vacuous area in the nuclear matrix during the course of apoptosis in vitro [35]. An overall synthesis may be that dissolution of the nuclear scaffold, that normally connects the chromatin, is coincident with a compartmentalization of PCNA and other nuclear matrix proteins, all of which leave chromatin strands unsupported and exposed in a vacuous area.

## Staggered, protracted death and intermittent secondary necrosis

**Fig. 4:**
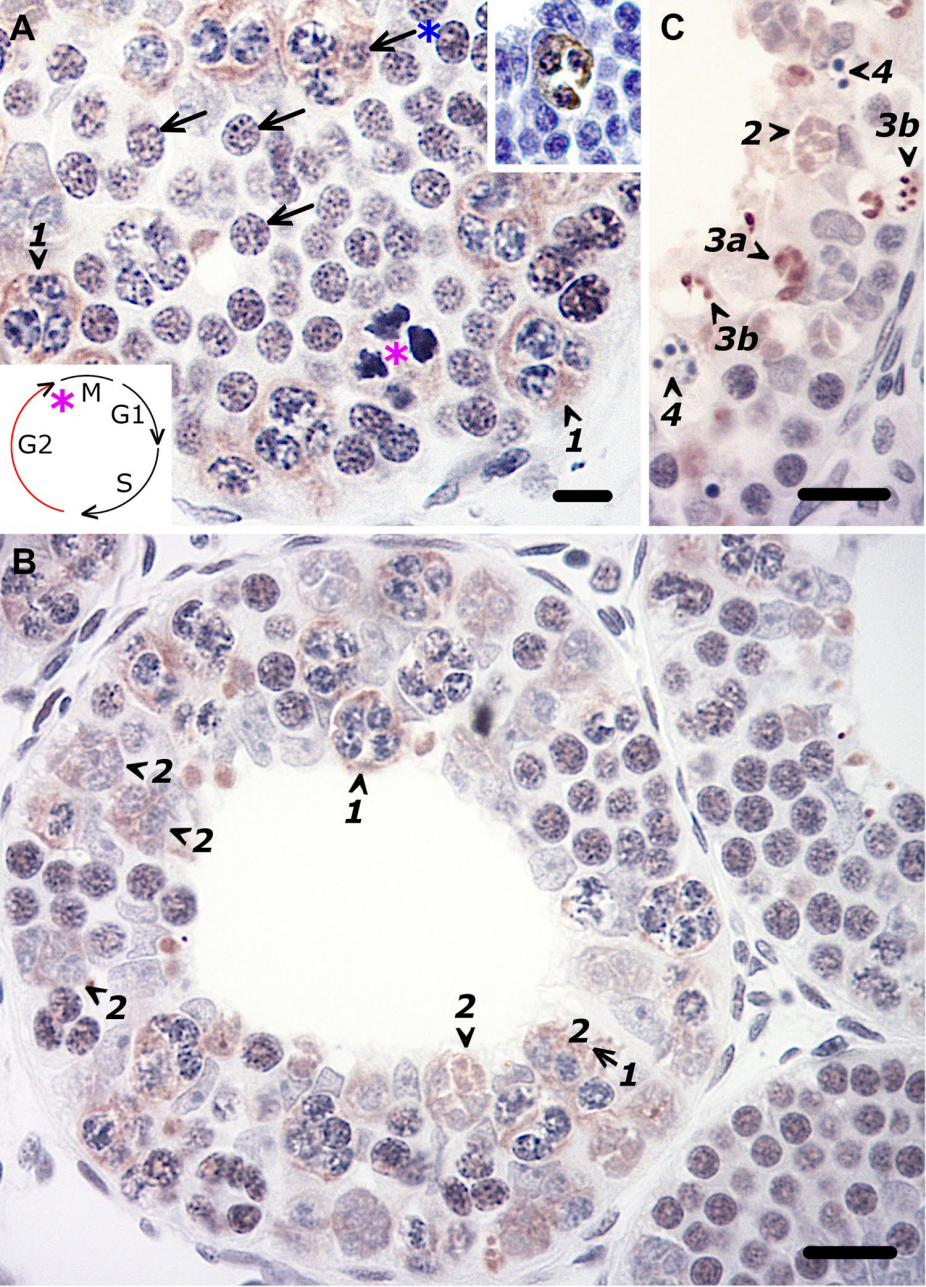
Differential PCNA labeling of the route of staggered, clustered spermatogonial deaths seen in contiguous G2-phase mature PrM cysts in the MNC testis condition. **A** A hallmark of these deaths affecting G2 spermatogonia (arrows) is the dark blue blotchy nucleus due to unscheduled excessively condensing chromatin, a development that is distinctly and anomalously PCNA-delineated (1)**.** The fortuitous sight of a lone still recognizable G2-phase spermatogonium (blue asterisked arrow) in a PCNA-delineated cluster confirms the passage into death while in G2. Note also anomalous PCNA-labelling of abortive mitosis (pink asterisk) as discerned from the frayed ends of the highly condensed chromatin threads. (Inset) The corresponding TUNEL-labeling of clustered deaths. **B** An adjoining turgid cyst immediately downstream shows many more PCNA-delineated clustered spermatogonial deaths plus further advances in the death process, e.g., coalesced spermatogonia now lacking basophilia and being fragmented (2), and Sertoli nuclei that have left their periluminal location and are now interspersed amongst the clusters of spermatogonia. **C** Severely germ cell-depleted cysts further downstream reveal the conclusion of the death process, namely the increasingly intense PCNA-labelling of the nuclear fragments that are also becoming smaller (3a → 3b). The appearance of pyknotic bodies (i.e., secondary necrosis, 4) is always coincident with the latter. Bar = 10 µm (Colour figure online)

In contrast to the immunomorphologically inimitable spermatogonial deaths around the time of exit from S-phase, entry into death while engaged in the G2‐M-phase passage is very different, not least because of ready visualization of excessively condensing chromatin strands and accompanying anomalous PCNA-labeling. As shown previously [30], G2-phase spermatogonia are readily discerned as showing an uneven light brown/globular appearance, a broad classification that also refers to the visualization of fine tortuous blue profiles in the nuclear architecture (i.e., increased separation of the chromatin into distinct threads, Fig. 4A). A light microscopically discerned anomaly in the latter chromatin configuration is the hallmark of these G2-phase deaths, namely a dark blue blotchy nucleus due to unscheduled excessively condensing chromatin. This aberration while in G2 is consistently and anomalously PCNA-immunodetected. The magnitude of these G2-phase cell death phenomena indicates a more synchronized, yet drawn-out course of events, most likely aided by opened/fused intercellular bridges that result in the clustering of dying spermatogonia, all while the cyst maintains normal turgidity and cellular organization (Fig. 4B). What seems like the chaotic cessation of the cyst’s expected spermatogenic advance (Fig. 4B), becomes meaningful when cognizant that the course of the clone’s demise is indeed systematic and in accordance with the wave-like, linear fashion of spermatogenic development in contiguous cysts. Thus, cysts immediately upstream display the early stages of the clustered death process that have reached an advanced stage, manifesting as secondary necrosis in cysts immediately downstream. For example, whereas a G2-cyst showing the initial stages of cell death reveals only a few PCNA-demarcated, highly basophilic clusters of spermatogonia (Fig. 4A), the adjacent cyst that is at a further stage of demise (Fig. 4B) shows the latter plus completely coalesced spermatogonia that now lack basophilia, and fragmentation of these clustered corpses. Accordingly, the severely germ cell-depleted cyst reveals the conclusion of apoptotic death and PCNA’s very different role there in the penultimate stage of apoptotic body formation (Fig. 4C). Remarkably though, such cysts may still contain a sparse population of normal looking spermatogonia. It is worthwhile to note the absence of secondary necrosis in the earlier stages (Fig. 4A, B) versus its scattered occurrence, even in a severely germ cell-depleted cyst, all of which explain the designation of phenomena here as staggered death, and the intermittent, and not massive, occurrence of apoptotic bodies.

The crisp PCNA-labelling of the late stages of apoptotic death are reminiscent of similar findings made about apoptotic cells in other tissues, including the in vivo carcinogen-exposed rat colon [36], regressed rat prostate [37] and human brain [38]. The novel findings though of intense PCNA-labelling of small fragments that is the penultimate stage of the pyknotic (apoptotic) bodies, indicate the involvement of PCNA in these late stages of apoptosis. These observations find support from earlier suggestions of the induction of replicative foci and of DNA repair during apoptotic death [34] and in vitro findings that nucleosomal fragments generated in late in apoptosis contain actively replicating DNA structures within their internal regions [39].

**Fig. 5:**
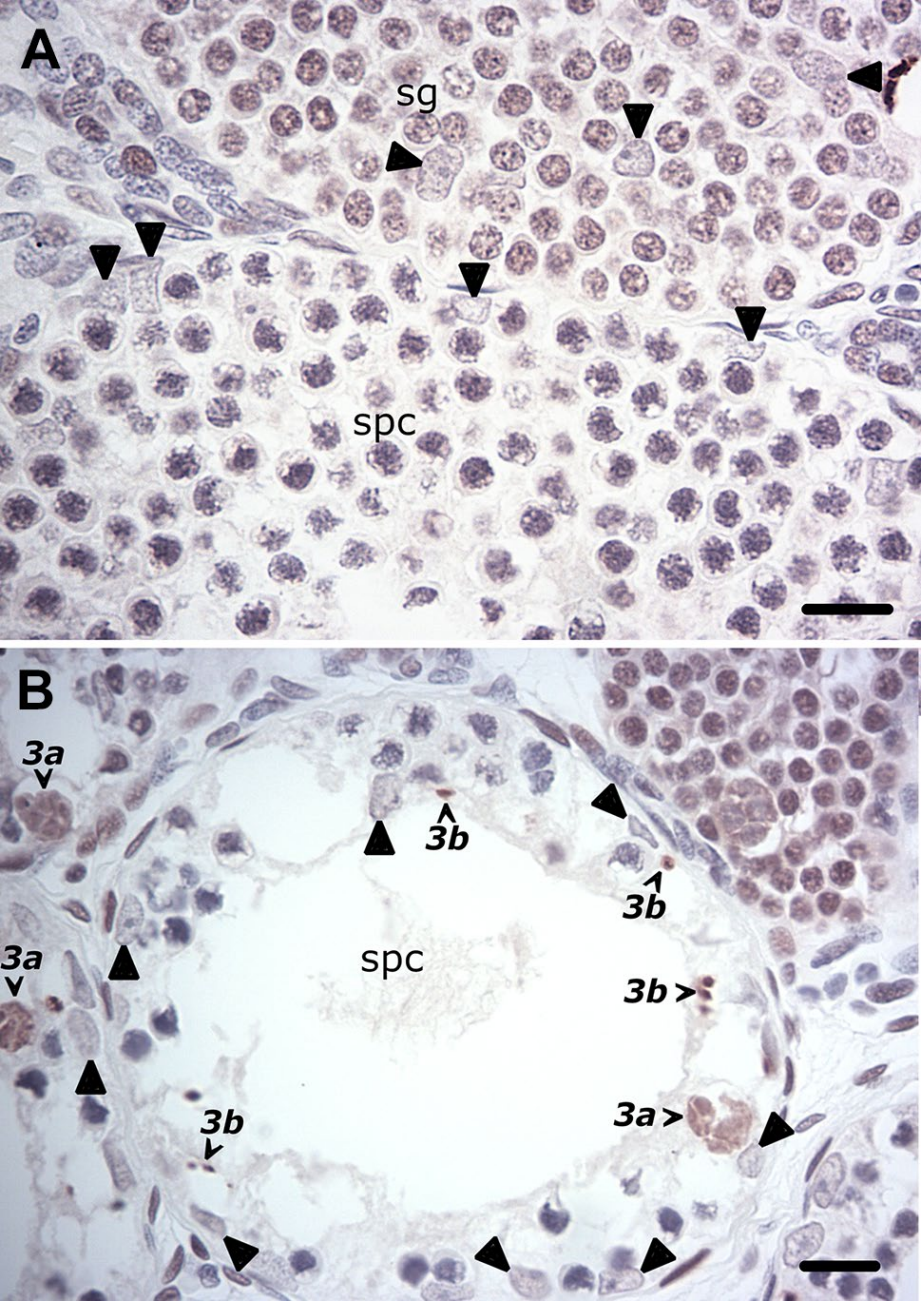
The abrupt cessation of PCNA immunoreactivity in spermatocyte cysts at the normal and perturbed mitosis–meiosis transition. **A** A cyst with a full complement of spermatocytes (spc) that is adjacent to a final-stage PrM cyst (sg) reveals quite diminished PCNA immunostaining. **B** A severely germ cell-depleted cyst that nevertheless still recovered from the earlier MNC wave of death and subsequently entered meiosis, is likewise completely devoid of PCNA-immunoreactivity despite the last remnants of PCNA-labeled clustered spermatogonial death and its fragments. Bar = 20 µm

The above spermatogenic stage-related PCNA immunostaining patterns are further validated by the abrupt cessation of immunoreactivity in spermatocyte cysts at both the normal (Fig. 5A) and MNC-disrupted (Fig. 5B) transition into meiosis.

**Fig. 6:**
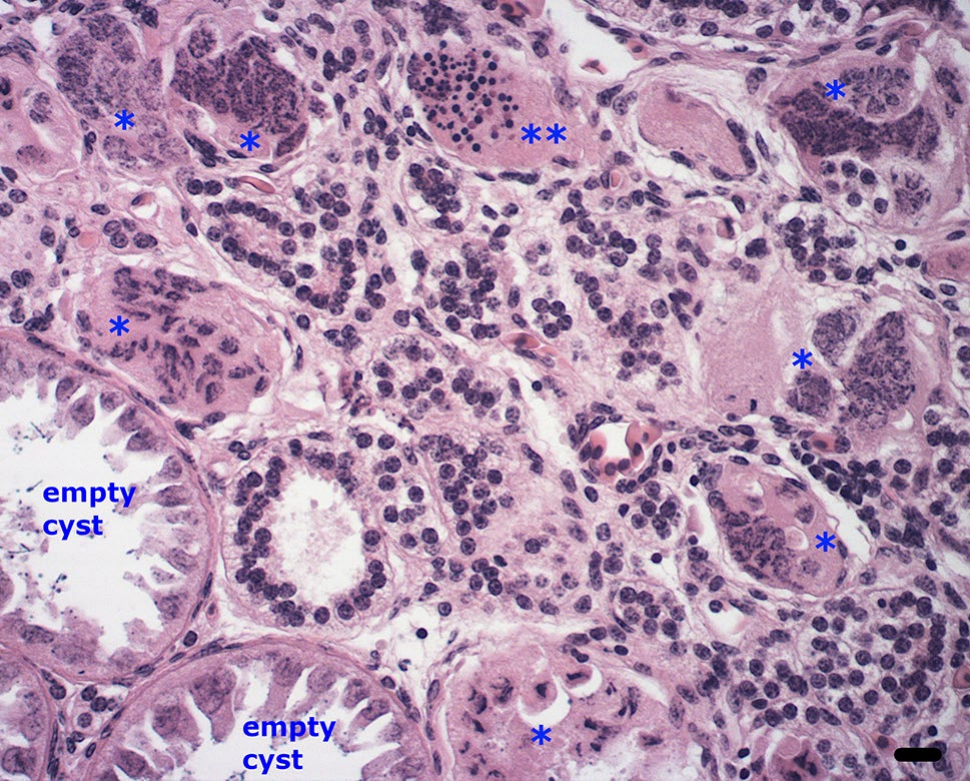
Hematoxylin and eosin-stained section showing the disposal of the empty cyst with its bulky Sertoli cell nuclei post-spermiation in the resorption zone of the thresher shark. The Sertoli cell nuclei are gradually dismantled and cyst wall dissolved (single asterisks), all of which culminate as massive secondary necrosis (double asterisks). Bar = 20 µm

An interesting corollary to the scattered secondary necrosis seen in severely affected mature PrM cysts is the massive, uniform secondary necrosis that occurs after the conclusion of spermatogenesis in the elasmobranch testis. After having completed spermatogenesis, the cysts discharge their fully differentiated spermatids, leaving the mature pole littered with emptied cysts that only contain Sertoli cells. The entire cyst’s Sertoli cell nuclei are stepwise fragmented in unison, all of which culminates in the display of uniform secondary necrosis (Fig. 6).

## Secondary necrosis: an outcome with a naïve phagocyte?

It is clear then that the elasmobranch testis may display massive secondary necrosis. However, the magnitude of the phenomenon clearly correlates with the cell type that needs to be phagocytically cleared. Given earlier notions that secondary necrosis is typically associated with delayed, non-availability or lower efficiency of phagocytosis [10, 14], these observations in the blue shark testis suggest that the staggered spermatogonial apoptotic death and associated intermittent secondary necrosis may well relate to the phagocytosis capacity of PrM cyst’s Sertoli cells that are essentially still functionally naïve at this juncture of elasmobranch spermatogenesis. It should be kept in mind that the elasmobranch spermatogonial clone’s own Sertoli cells are assembled anew at the start of each round of spermatogenesis [30] and are therefore functionally still immature [40] at the time they are faced with a voluminous apoptotic cargo load.

Lastly, the non-involvement, during massive cyst breakdown, of nearby professional phagocytes from the testis-affixed bone marrow equivalent of elasmobranchs (i.e. epigonal organ) underscores the importance of the Sertoli cell as the preferred phagocyte in these primitive vertebrates, as is also the case in advanced vertebrates. The mammalian Sertoli cell with its wide-ranging repertoire of anti-inflammatory functions [41], has been designated a specialized phagocyte [42]. It is therefore concluded that what appears as wide-scale apoptotic death and prolonged phagocytic elimination of corpses during ongoing spermatogenic development in other cyst stages is probably due to the lower efficiency of phagocytosis of one of the elasmobranch’s specialized phagocytes, namely its Sertoli cell.

## Data Availability

None.
